# Annotations

**Published:** 1892-12-03

**Authors:** 


### Annotations.

No regular reader of The Hospital will charge us with being violently pro-Pasteurian. We have always insisted that Pasteur's antirabic treatment, like Koch's antiphthisis inoculation, demands further proof. But what does not need any further demonstration whatever is the extreme importance and value of modern bacteriological research, and the high character and admirable competence of British researchers. On July 1st, 1889, the British leaders of medicine and science asked from the platform of the Mansion House for £120,000, wherewith to build and endow a British Institute of Preventive Medicine. To-day, at the end of three and a-half years, they are asking still. To say the least, the public has not been in a hurry to show any enthusiastic confidence in the leaders of medicine. And yet, to those who know, the leaders of medicine at this moment are among the very worthiest specimens of Englishmen our proud country has ever produced. Honest, thorough, full of splendid common sense, broadly cultured, and imbued with the noblest ideals, they constitute a band of men whom any age might deem it honourable to honour. If half-a-dozen medical men of considerable name had conspired with Mr. Stead to proclaim the greatness of Mattei's so-called cancer cure, £50,000 might have been raised in a few months. But when scores of the most distinguished names in medicine and science unite to ask help in the prosecution of truth and practical scientific research, it has not been found possible to raise fifty thousand pounds in three and a-half years. Even of the sum that has been raised, the doctors themselves have subscribed almost the larger proportion. Those doctors who have given the money have no private ends to gain; they have already established their reputation, and some of them have secured fortune as well as fame. We have always taken the line of entire independence on the subject of Pasteurism and Kochism, and we have not hesitated to differ strongly from even the most eminent of our professional brethren, when it seemed to be our duty to do so. It is in exactly the same spirit of independence that we make remonstrance and appeal to all thoughtful Englishmen for a practical vote of confidence in the leaders of science and medical practice, who have identified themselves with the British Institute of Preventive Medicine. The public has not understood the merits of the case. If it had, the money would have been raised long ago. But, put simply, the response to the appeal amounts to neither more nor less than a vote of confidence in British leaders of science, medicine, and surgery. Does the public think those leaders worthy of confidence to the extent of £120,000? If it does—and how can it do otherwise?—let it promptly pay down the money! If it does not, why then, so much the worse for the public's reputation for culture and sense.

**A Vote of Confidence for Modern Medicine.**

Those who take notice are now beginning to understand that margarine is no substitute for butter. Not long ago an interesting experiment clearly demonstrated the fact. The inmates of a certain blind asylum had margarine supplied to them instead of butter. At first nothing was said: but in a little time complaints began to be made that the butter seemed to be losing its flavour: by and bye, first one and then another gave up taking the margarine, which they supposed to be butter. At the end of a few months not one inmate cared any longer for butter; they all declared they had lost their taste for it. Of course, as the patients were all blind they could not see that they were eating margarine, and to the end of the experiment they were all of opinion that it was "butter" which they had been eating, and which they had for some reason simultaneously "got tired of." The Margarine Act has amply proved its necessity and utility during the past four years. The chief complaint that honest people and people of "taste" have to make is that it has not been administered with sufficient sternness. It is something, no doubt, to have had nearly fifteen hundred prosecutions in England alone, and that close upon thirteen hundred of them have resulted in convictions; but it is a pity that the manufacturers are the persons who mostly escape. We have no objection to the sale of margarine as "margarine;" but to have it palmed off as butter is a wicked fraud, which demands vigorous repression. The English man of business is a worse offender than the Welsh in the substitution of margarine for butter, and much worse than the Scotch or Irish. The Irish, indeed, have experienced extremely few prosecutions. Perhaps they have not been sharply enough looked after. The chief criminals in Great Britain have been the tradesmen of large towns. Country people know butter when they taste it, and they cannot, therefore, be so easily imposed upon. Liverpool has reached the "bad eminence" of being the worst city in the kingdom in the margarine imposture. The successful convictions in Liverpool during the four years 1888, '89, '90, and '91 numbered 319: those in London amounted to no more than 218; that is to say, Liverpool is about ten times worse than London in this matter. We do not say that margarine is positively unwholesome, but it is at best sorry stuff, much inferior to beef or bacon dripping, and quite unworthy even to be compared with good butter. What a pity it is that farming is not done scientifically in this country! If it were, there is no reason whatever why excellent butter should not be supplied all the year through at ninepence a pound.

**Four Years of Margarine.**

A correspondent writes to complain of the behaviour of a nurse. Many correspondents have written at various times to complain of the behaviour of nurses. The nurse wishes to go out for an hour every day; the patient's friends are willing to allow her to go out, "when it is convenient." The rule of "convenience" has worked out in practice in such a way that the nurse has been able to get a walk six days out of nine. Our correspondent seems to think this reasonably good; the nurse, on the other hand, does not see the matter in that light, and has therefore been, so it is said, "impertinent." The evidences of friction between nurses and their employers are accumulating fast. Now this is an evil which should be put an end to. There are rules in existence which, if faithfully carried out on both sides, would certainly prevent friction. But rules are like machinery, and will not work automatically. They need steam power, like machinery; and oil for their wheels and joints, like machinery. The rules ought to be, and must be, interpreted and worked by means of unselfish devotion on the part of her employers, and kindly consideration on the part of the nurse. Undoubtedly a nurse should get out an hour every day; but it may happen now and again that she must give her going out hour to her patient. If this should be so, let the hour be given ungrudgingly and loyally, remembering always that the helpless patient is the chief consideration. On the other hand, if the one absolutely necessary hour is taken from the nurse sometimes, it should be given back again as soon as possible. The employer has no right to "steal" the nurse's "health hour." And the theft would certainly not be profitable to the patient. If the nurse is to do her duty thoroughly and efficiently she must preserve her own health, and this cannot be done at all unless at least an hour every day is spent in the open air. "Give and take" is the rule in nursing, as in all other relations of life.

